# Early decrease in carotid plaque lipid content as assessed by magnetic resonance imaging during treatment of rosuvastatin

**DOI:** 10.1186/1471-2261-14-83

**Published:** 2014-07-14

**Authors:** Ruixue Du, Jianming Cai, Xue-Qiao Zhao, Qing-Jun Wang, Dan-Qing Liu, Wen-Xiu Leng, Peng Gao, Hong-Mei Wu, Lin Ma, Ping Ye

**Affiliations:** 1 Department of Geriatric Cardiology, Chinese PLA General Hospital, No. 28, Fuxing Road, Beijing 100853, China; 2 Department of Radiology, Chinese PLA General Hospital, No. 28, Fuxing Road, Beijing 100853, China; 3 Department of Medicine, Division of Cardiology, University of Washington, Seattle, WA, USA

**Keywords:** Atherosclerosis, Plaque lipid content, Statin, Magnetic resonance imaging

## Abstract

**Background:**

Statin therapy has shown to deplete atherosclerotic plaque lipid content and induce plaque regression. However, how early the plaque lipid depletion can occur with low-density lipoprotein cholesterol (LDL-C) lowering in humans *in vivo* has not been fully described.

**Methods:**

We enrolled 43 lipid treatment naïve subjects with asymptomatic carotid atherosclerosis and LDL-C ≥ 100 and ≤ 250 mg/dl. Rosuvastatin 5–20 mg/day was used to lower LDL-C levels to < 80 mg/dl. Lipid profile and carotid MRI scans were obtained at baseline, 3, 12, and 24 months. Carotid plaque lipid-rich necrotic core (LRNC) and plaque burden were measured and compared between baseline and during treatment.

**Results:**

Among the 32 subjects who completed the study, at 3 months, an average dose of rosuvastatin of 11 mg/day lowered LDL-C levels by 47% (125.2 ± 24.4 mg/dl vs. 66.7 ± 17.3 mg/dl, *p* < 0.001). There were no statistically significant changes in total wall volume, percent wall volume or lumen volume. However, LRNC volume was significantly decreased by 7.9 mm^3^, a reduction of 7.3% (111.5 ± 104.2 mm^3^ vs. 103.6 ± 95.8 mm^3^, *p* = 0.044). Similarly, % LRNC was also significantly decreased from 18.9 ± 11.9% to 17.9 ± 11.5% (*p* = 0.02) at 3 months. Both LRNC volume and % LRNC continued to decrease moderately at 12 and 24 months, although this trend was not significant.

**Conclusions:**

Among a small number of lipid treatment naïve subjects, rosuvastatin therapy may induce a rapid and lasting decrease in carotid plaque lipid content as assessed by MRI.

**Trial registration:**

ClinicalTrials.Gov numbers NCT00885872

## Background

Atherosclerosis alone is thought to be a relatively benign disease and progresses with aging; however, it is frequently complicated by acute thrombosis, usually triggered by the rupture or erosion of an atherosclerotic plaque which is determined by plaque morphologic characteristics, local composition and inflammation [1,2]. Plaque rupture or erosion can lead to major cardiovascular events such as acute coronary syndromes and strokes. The cardiovascular events reduction with statin therapy demonstrated in the past 2 decades is most likely induced by improved plaque stability that is determined by lipid core size, fibrous cap thickness and the level of inflammatory infiltrates and activity.

Development of high-resolution magnetic resonance imaging (MRI) techniques in recent years has made it possible to directly assess plaque composition. Numerous studies have shown that MRI creates high contrast for internal features of plaques and that the combined information from multiple contrast weightings is critical for distinguishing all plaque components [3]. The accuracy and reproducibility of carotid MRI has been extensively validated, using histological analysis of lesions [4,5]. Thus, carotid MRI can accurately assess plaque tissue contents.

Previous studies have demonstrated that low-density lipoprotein cholesterol (LDL-C) lowering with statin induced plaque regression [6,7] and plaque lipid content reduction [8,9]. However, how early the plaque lipid depletion can occur with intensive LDL-C lowering in humans *in vivo* has not been fully described.

Thus, we designed and conducted a prospective study, Rosuvastatin Evaluation of Atherosclerotic Chinese Patients, (REACH study, NCT00885872), to test the hypothesis that statin therapy induces rapid plaque lipid depletion as assessed by MRI.

## Methods

### Research subjects

**Figure 1:**
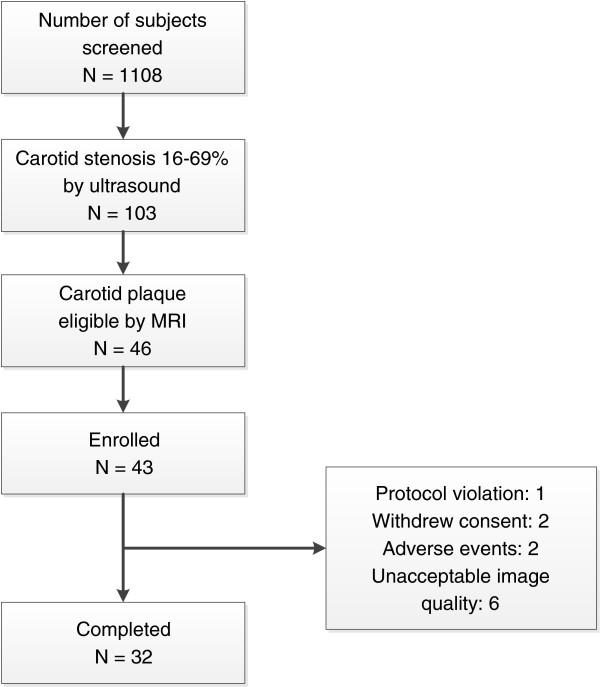
REACH study recruitment, enrollment and completion.

Subjects were recruited and enrolled to the REACH study if they met the following inclusion criteria: 1) age 18 to 75 years; 2) carotid stenosis 16 - 69% by ultrasound; 3) the maximum wall thickness ≥ 3 mm, an intact fibrous cap as assessed by MRI; 4) LDL-C ≥ 100 mg/dl and ≤ 250 mg/dl; 5) triglycerides ≤ 353 mg/dl; and 6) no history of statin or other lipid treatment. 7) The patients who were willing to be enrolled have to remain on the low cholesterol dietary before the screening. Exclusion criteria were: 1) uncontrolled hypertension; 2) severe heart disease; 3) peripheral arterial disease; 4) liver disease; 5) renal dysfunction; 6) hypothyroidism; 7) uncontrolled hyperglycemia with an HbA1c > 9%; 8) familial hypercholesterolemia; 9) history of any lipid-lowering agents.As shown in Figure 1, among 1108 subjects screened by ultrasound, 103 were identified to have stenosis of 16 - 69% and suitable for carotid MRI scans. 46 subjects were confirmed to meet the above MRI requirements. Of the 46 subjects, 43 qualified for lipids and without any liver and renal abnormalities and enrolled in the REACH study. During the study, 1 patient was withdrawn for protocol violations, 2 withdrew consent, 2 experienced adverse events (one for increases in alanine aminotransferase and aspartate aminotransferase, the other for increases in creatine kinase), and 6 were excluded from the final analyses for poor image quality or missing one or more scans. Thus, 32 subjects completed the study with matched serial MRI scans and acceptable image quality.

The REACH study was carried out at the People Liberation Army (PLA) General Hospital (Beijing, China) between March 29th, 2009 (first patient enrolled) and February 26th, 2012 (last patient completing the study). All procedures were performed according to the Declaration of Helsinki, and the study protocol was approved by the PLA General Hospital’s institutional review board. All patients provided a written informed consent.

### Statin therapy and follow-up

All subjects received an initial treatment of rosuvastatin 10 mg daily. Dose adjustment was performed after 4 weeks according to the Chinese guidelines for the prevention and treatment of adult dyslipidemia [10]. If LDL-C levels were ≥ 80 mg/dl, or if HDL-C were < 40 mg/dl, rosuvastatin dose was increased to 20 mg. If LDL-C levels < 80 mg/dl, rosuvastatin dose was maintained at 10 mg. Rosuvastatin dose was decreased to 5 mg when LDL-C levels were < 50 mg/dl. The dose determined at 4 weeks was maintained during the rest of the 24-month.

After the study initiation, regular clinic visits were scheduled every 1 to 3 months over the 24-month follow-up period. At each visit, vital signs, physical examination, dietary counseling, adverse events monitoring and medication compliance calculation were performed. Serum lipids, liver functions, kidney functions and muscle enzymes were assessed at 3, 12, and 24 months.

### Magnetic resonance imaging protocol

High-resolution and multicontrast bilateral carotid MRI scans were performed using a 3.0-T MRI scanner (Signa Echo Speed, GE Healthcare, Waukesha, WI, USA) at baseline, and at 3, 12, and 24 months. All follow-up MRI scans were performed using the same imaging protocol and carefully matched for scan coverage using the carotid bifurcation as an internal landmark. An index artery with the worse maximum wall thickness was identified at the baseline scan.

A standardized protocol was used to obtain 3-dimensional time-of-flight (TOF), proton-density-weighted (PDW), T2-weighted (T2W) and T1-weighted (T1W) images. Twelve axial images, centered at the bifurcation of the index artery, were acquired with a 2-mm slice thickness for a total longitudinal coverage of 24 mm without gap. Gadolinium contrast material (Magnevist at 0.1 mmol/kg) was then administrated intravenously and the T1-weighted scan was repeated 5–7 minutes after contrast administration. Total acquisition time for all images was approximately 40 minutes.

### MRI analysis of plaque morphology and composition

All images were analyzed by experienced reviewers at IBMarker Company, Seattle, WA, USA. An experienced reviewer matched the axial images from all 4 time points according to the distance from the carotid bifurcation. Then, two experienced reviewers, who were blinded to the order of MR scans, evaluated and interpreted all images with matched locations of the index artery by reaching a consensus opinion. This image review process was blinded to laboratory results and clinical course.

Using a custom-designed image analysis tool (CASCADE, IBMarker Company, Seattle, WA, USA), the lumen and outer wall boundaries were identified [4,11]. The total vessel area and lumen area (LA) were quantified by placing contours around the outer wall boundaries and the lumen of the carotid artery, respectively. Wall area (WA) was calculated by subtracting the lumen area from the total vessel area. Wall, lumen volumes and total vessel volumes were obtained by adding the areas across slices and multiplying by the slice thickness (2 mm). Percent wall volume (PWV), which is similar to percent atheroma volume described in the IVUS literature, was calculated as: (wall volume/total vessel volume) × 100%.

Lipid-rich necrotic core (LRNC) was identified and automatically quantified using previously published histologically-validated MRI criteria [4,5,11]. Specifically, LRNC was identified using multicontrast weightings plus post-contrast T1W images. LRNC usually appears as isointense to hyperintense on the TOF and pre-contrast T1W images, has variable signal intensity on PDW and T2W images, and has no or slight contrast enhancement compared with the surrounding tissue on post-contrast T1W images. Total LRNC volume was measured by multiplying the slice thickness by the sum of the LRNC areas. The proportion of LRNC relative to the wall volume (% LRNC) was calculated as: (LRNC volume/wall volume) × 100%. Both LRNC volume and % LRNC were calculated using images containing LRNC.

According to previous studies, the reproducibility of measuring plaque burden (ICC ranging from 0.96-0.99) and LRNC size (ICC ranging from 0.94-0.99) with automatic analysis is good to excellent [4,8,12].

### Endpoints

The primary endpoint was the carotid plaque lipid content presented as LRNC volume and %LNRC as measured by MRI. The secondary endpoint was the plaque burden presented as PWV.

### Statistical analysis

The sample size was estimated according to previous similar study [8]. For the change in % LRNC at baseline, a sample size of approximately 26 patients was required for 90% power and a 2-sided level of 0.05 to detect an expected change of 2.7%, assuming an SD range from 2.5% to 5.3%. If approximately 25% of patients discontinued from the study, then 35 patients allocated to study medication would result in 26 patients completing the study, which would provide sufficient power to assess the primary end points.

Descriptive statistics are presented as mean ± SD. Changes over time in lipids were compared to baseline using the Wilcoxon’s signed-rank test (non-normally distributed variables) or the paired *t*-test (normally distributed). Changes over time in plaque measurements were compared to baseline using the paired *t*-test when data presented normally distributed. Otherwise, data were logarithmically transformed to normalize their distributions. Associations test of plaque lipid content changes were assessed using the Spearman rank correlation coefficient. Analysis was carried out using SPSS 17.0 (SPSS Inc. Chicago, IL, USA). A *p*-value < 0.05 was considered statistically significant.

## Results

### Patient baseline characteristics

Baseline demographic characteristics for the 32 subjects who completed the trial are summarized in Table 1. The mean age was 61 years and body mass index was 24.5 kg/m^2^. Seventy-eight percent of subjects were male, 78% had hypertension, 31% were diabetics, 9% had coronary artery disease and 31% had history of cerebrovascular disease.

**Table 1:** Patient baseline characteristics

**Characteristics**	Patients (n = 32)^*^
Age, mean (SD), y	60.8 ± 9.1
Male	25 (78.1%)
Body mass index, mean (SD)^†^	24.5 ± 2.7
History of hypertension	25 (78.1%)
History of diabetes mellitus	10 (31.3%)
History of coronary heart disease	3 (9.4%)
History of cerebrovascular disease	10 (31.3%)
Current smoking	3 (9.4%)
Concomitant medications	
Aspirin	10 (31.3%)
Antihypertensive	25 (78.1%)
Hypoglycemic agents	10 (31.3%)

^*^Data are expressed as number (%) unless otherwise specified.

^†^Calculated as weight (in kilograms) divided by the squared height (in meters).

### Changes in lipid levels under rosuvastatin therapy

Rosuvastatin 5–20 mg/day was initiated at the study entry and dosage adjustment was made at 4 weeks to achieve LDL-C levels < 80 mg/dl. The mean dose was 11 mg/day during the remaining of the study. Lipid levels during the 2 years of rosuvastatin therapy were presented in Table 2. At 3 months, LDL-C levels were significantly reduced by 47% (125.2 ± 24.4 mg/dl vs. 66.7 ± 17.3 mg/dl, *p* < 0.001). HDL-C levels were increased by 4% (49.2 ± 14.7 mg/dl vs. 51.2 ± 11.8 mg/dl, *p* = 0.084). Triglycerides were lowered by 27% (142.3 ± 67.1 mg/dl vs. 103.3 ± 41.6 mg/dl, *p* < 0.001).

**Table 2:** Lipid levels during the 24-month therapy

	**Baseline**	**3 Months**	**12 Months**	**24 Months**
Total cholesterol (mg/dl)	200.8 ± 29.8	135.8 ± 24.7^*^	135.1 ± 36.0^*^	139.8 ± 24.4^*^
LDL-cholesterol (mg/dl)	125.2 ± 24.4	66.7 ± 17.3^*^	65.5 ± 17.0^*^	69.8 ± 16.6^*^
HDL-cholesterol (mg/dl)	49.2 ± 14.7	51.2 ± 11.8	52.9 ± 14.2^†^	53.8 ± 14.8^*^
Triglycerides (mg/dl)	142.3 ± 67.1	103.3 ± 41.6^*^	110.6 ± 50.4^†^	98.7 ± 48.2^*^

Data are presented as mean ± SD. *LDL* = low-density lipoprotein; *HDL* = high-density lipoprotein. ^*^p < 0.001 when compared to baseline. ^†^*p* < 0.05 when compared to baseline.

### Effects of rosuvastatin on plaque lipid content

At 3 months, LRNC volume was significantly decreased by 7.9 mm^3^, a reduction of 7.3% (111.5 ± 104.2 mm^3^ vs. 103.6 ± 95.8 mm^3^, *p* = 0.044). % LRNC was significantly decreased from 18.9% ± 11.9% to 17.9% ± 11.5% (*p* = 0.02) as shown in Table 3.

**Table 3:** Changes in LRNC and carotid arterial wall burden

	**Baseline**	**3 Months**	**12 Months**	**24 Months**
LRNC, mm^3^	111.5 ± 104.2	103.6 ± 95.8^*^	101.7 ± 93.4^*^	97.7 ± 91.5^*^
LRNC%	18.9% ± 11.9%	17.9% ± 11.5%^*^	17.5% ± 11.2%^*^	16.7% ± 11.3%^*^
Wall volume, mm^3^	533.3 ± 208.9	523.1 ± 196.3	521.0 ± 194.2	528.0 ± 198.9
Lumen volume, mm^3^	522.1 ± 246.7	514.0 ± 234.2	512.1 ± 245. 6	537.6 ± 265.0
PWV, %	51.0% ± 8.2%	50.8% ± 8.5%	50.9% ± 9.0%	50.3% ± 8.6%

Data are presented as mean ± SD. Changes over time in plaque measurements were compared to baseline using the paired *t*-test (LRNC, LRNC % and lumen volume were logarithmically transformed to normalize their distributions). *LRNC* = lipid-rich necrotic core; % LRNC = proportion of LRNC relative to the wall area; *PWV* = percent wall volume. ^*^P < 0.05 when compared to baseline.

**Figure 2:**
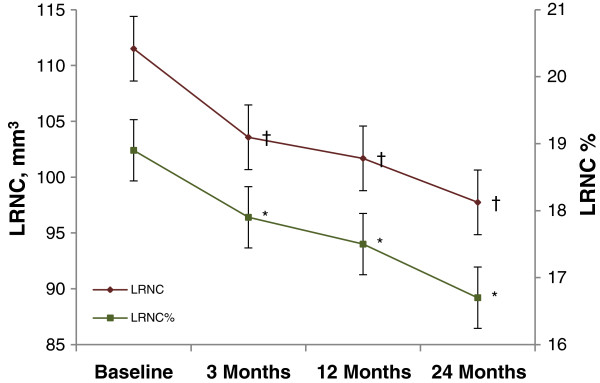
**Reduction in LRNC and % LRNC during the 24 months of rosuvastatin therapy.** The time course of plaque LRNC reduction over 24 months suggested that LRNC (red rhombuses) significantly decreased by 7.9 mm3, a reduction of 7.3% at 3 months, and continue to decrease moderately at 12 and 24 months. ^†^*p* < 0.05 when compared to baseline. There were no statistically significant differences when LRNC at 12 months compared to that at 3 months, and at 24 months vs. at 12 months. The decrease of % LRNC (green squares) displayed the same trend. ^*^p < 0.05 when compared to baseline. Bars around the estimates are standard error bars. LRNC = lipid-rich necrotic core.

**Figure 3:**
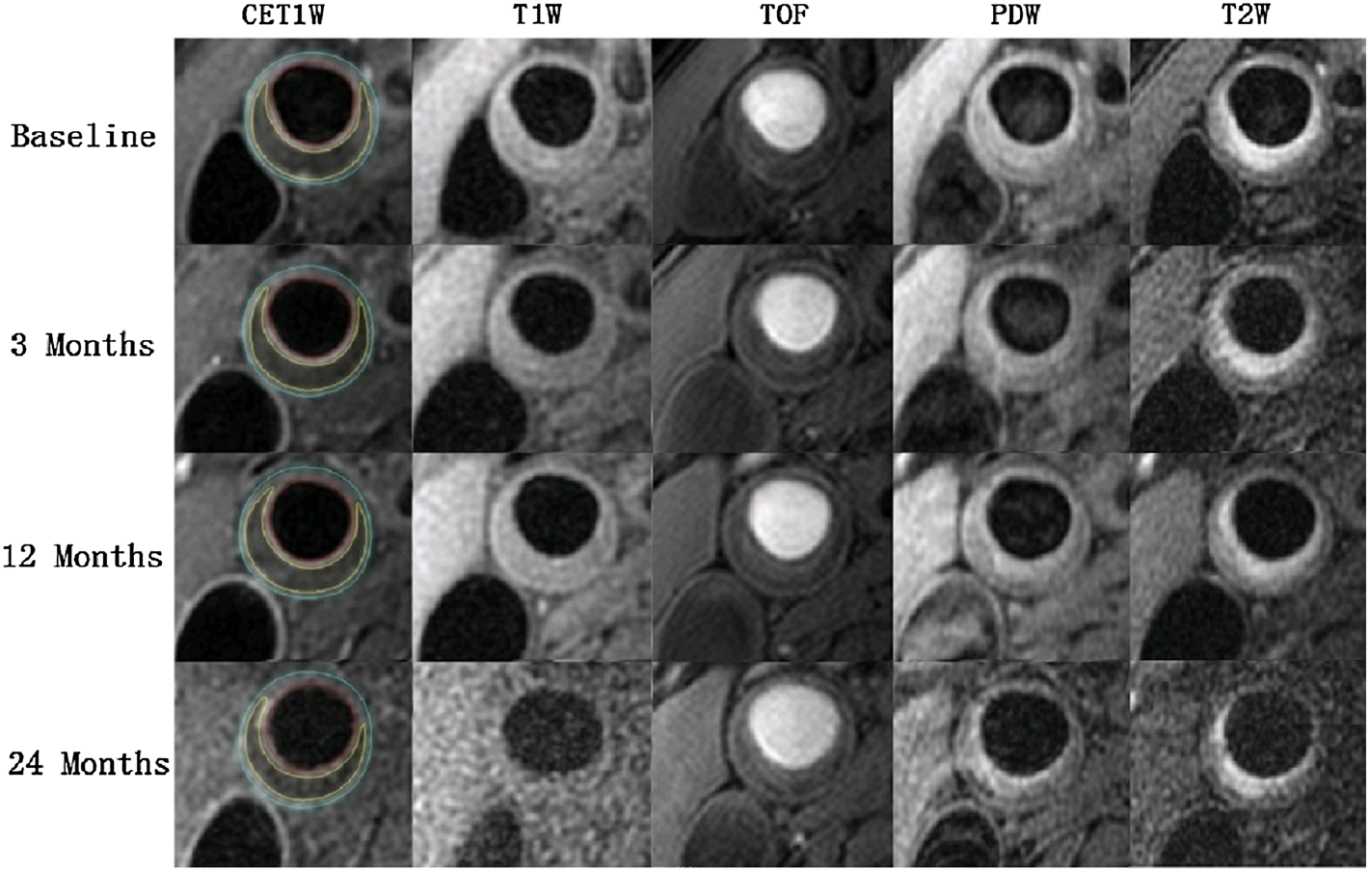
**Representative MRIs showing plaque lipid depletion in right common carotid artery over 2 years.** The lumen in red, outer wall boundary in blue and lipid content in yellow of the carotid artery were identified and outlined in post-contrast T1W images. LRNC demonstrate isointense on pre-contrast T1W images and TOF, and was detected as non-enhanced areas (relative to surrounding tissues) on CET1W images with the corresponding pre-contrast T1W images used as reference. Regression in LRNC at the same location was found between the baseline, 3 months, 12 months and 24 months MRI scans. MRI = magnetic resonance imaging; LRNC = lipid-rich necrotic core; TOF = time-of-flight; T1W = T1-weighted; T2W = T2-weighted; PDW = proton-density-weighted; CET1W = contrast enhanced T1-weighted.

The time course of plaque LRNC reduction over 24 months is illustrated in Figure 2 and described in Table 3. Following the initial significant reduction at 3 months, LRNC volume continued to decrease moderately at 12 and 24 months. However, there were no statistically significant differences when LRNC at 12 months compared to that at 3 months, and at 24 months vs. at 12 months. The decrease in % LRNC displayed the same trend.Among the 32 subjects who completed the study, 68.8% showed any decrease in LRNC volume and 62.5% had any decrease in % LRNC. Figure 3 shows a representative MRI of plaque lipid core regression over 2 years. However, there was no subject demonstrated a completed depletion of plaque lipid content over the 24 months. There were 31.2% of subjects showed no change or increase in LRNC.

**Figure 4:**
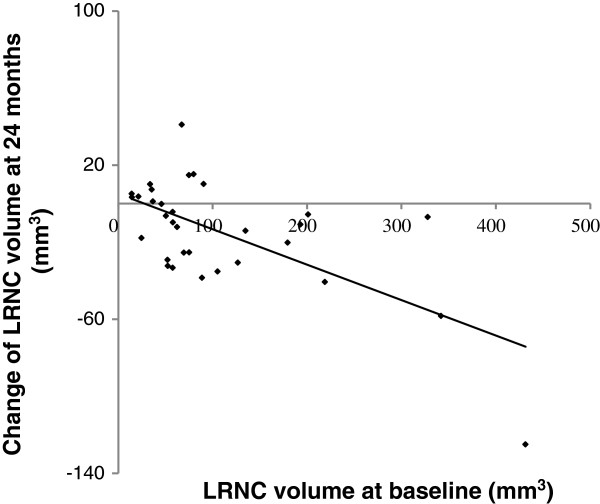
**Associations between changes of LRNC volume at 24 months and LRNC volume at baseline.** Spearman rank correlation showed that magnitude of LRNC decrease over 24 months was significantly correlated with the LRNC volume at baseline (r = - 0.53, *p* = 0.002).

Furthermore, magnitude of LRNC decrease over 24 months was significantly correlated with the LRNC volume at baseline (r = - 0.53, *p* = 0.002) as shown in Figure 4.

### Effects of rosuvastatin on carotid plaque burden

As shown in Table 3, during the 2 years of rosuvastatin therapy, PWV and the total wall volume slightly decreased, and lumen volume slightly increased. However, none of these changes reached statistical significance.

Nevertheless, the decrease in total wall volume was statistically correlated with reduction in LRNC at 3 (r = 0.42, *p* = 0.039), 12 (r = 0.44, *p* = 0.027) and 24 months (r = 0.45, *p* = 0.024). The decrease in PWV over 24 months was statistically associated with reduction in LRNC (r = 0.40, *p* = 0.005).

## Discussion

It is well documented that major determinants of plaque rupture are cellular and lipid composition and associated inflammation [1,2] while specific plaque features associated with propensity for disruption include higher lipid [2,13,14] and macrophage content [2,15-17]. These high-risk plaque features have been pursued as therapeutic targets and measurable marker for plaque stabilization [18,19].

In addition to animal studies showed statin therapy let to plaque lipid depletion from the existing plaque [20,21], in a case–control study of patients randomized to 3 months of pravastatin treatment or placebo prior to carotid endarterectomy, Crisby et al. provided histological evidence of plaque lipid content reduction with therapy [22]. The present study demonstrated that carotid plaque lipid content (LRNC volume and % LRNC) measured by MRI decreased significantly at 3 months with moderate-dose rosuvastatin (11 mg/day) lowering LDL-C levels by 47% in a group of lipid treatment naïve subjects. Our study provides *in-vivo* verification of rapid plaque lipid depletion. Furthermore, taken together with a recent published study [23] showing that intensified statin therapy (atorvastatin 80 mg) was associated with rapid reduction in vascular inflammations determined by fluorodeoxyglucose-positron emission tomography, these findings may indicate evidence of an early on-set of plaque stabilization via a rapid reduction in arterial inflammation and a rapid plaque lipid depletion with statin therapy.

After a rapid and statistically significant plaque lipid depletion seen at 3 months in our study, carotid plaque lipid content continued to decrease moderately and no subject achieved completed plaque lipid depletion over 24 months of therapy. However, Carotid Plaque Composition by MRI during Lipid-lowering (CPC) study [9] showed a statistically significant reduction in plaque lipid content at 12 and 24 months compared to baseline and 11% lower frequency of subjects with measurable LRNC over 3 years. There are several important differences between the present study and CPC: (1) Chinese treatment naïve subjects vs. Caucasian subjects with lipid treat history < 1 year; (2) lower LDL-C (125 mg/dl vs. 163 mg/dl) and triglycerides (142 mg/dl vs. 202 mg/dl) levels at baseline in our study subjects; (3) higher LRNC volume (111.5 mm^3^ vs. 60.4 mm^3^) and % LRNC (18.9% vs. 14.2%) in present study; (4) statin alone therapy vs. 2/3 of subjects treated with combination lipid therapy. Saam et al. reported significant differences in LRNC area between Chinese and American patients with symptomatic carotid diseases [24]. A large LRNC at baseline and treatment naïve status in our study subjects may play a partial role in the significant rapid plaque lipid depletion. However, the lower dose of rosuvastatin used in current study might be related to the moderated lipid depletion. Further studies are needed to examine the plaque response to therapy in different ethnic groups, population with different lipid abnormalities and different magnitude of plaque lipid depletion associated with intensity of LDL-C lowering.

The application of MRI in prospective serial studies of human atherosclerosis has become more widespread because it is noninvasive, not involve ionizing radiation, superior to other imaging modalities in discriminating tissue contrast, and highly reproducible [25,26]. Prospective MRI studies of carotid lesions [27-29] offer compelling evidence that high-risk plaque features including a large LRNC, strongly associate with clinical cerebrovascular events in patients with asymptomatic 50%-79% carotid stenosis. The high-risk plaque features are also seen in less severe stenosis. In an MR angiography study of carotid arteries with 0% stenosis, LRNC was present in 67.4% of nonocclusive lesions [30]. Importantly, our study showed that plaques with a larger LRNC are more likely to deplete (r = - 0.53, *p* = 0.002) in response to a moderate-dose of rosuvastatin therapy. These data indicate that plaques with large LRNC are associated with a significantly higher risk of plaque rupture and also more likely to respond to statin therapy and suggest a potential mechanism of statin therapy in plaque stabilization leading to a lower risk for future ischemic events. On the other hand, despite a similar LDL-C lowering during the study, 31.2% of subjects showed plaque LRNC no change or increase. The clinical importance of persistent presence of plaque LRNC and other risk features with LDL-C lowering requires to be established in larger prospective clinical trials.

Although PWV and total wall volume was decreased and lumen volume was increased non-significantly over two years, these results were consistent with that seen in ORION [8]. Furthermore, the total wall volume reduction was significantly associated with plaque lipid content decrease as indicated in CPC [9]. Considering the natural increase of carotid plaque, the results indicate that rosuvastatin may halt the natural progression of carotid atherosclerotic plaques.

We recognize the limitations of the current study. Firstly, there was no control group since it is not ethically acceptable to study subjects with established carotid atherosclerosis without therapy. Although we performed the MRI analysis by an independent review group blinded to all clinical information, lab results and MRI scan time points, it may reduce the power to detect differences in relevant plaque endpoints. And we cannot exclude the possibility that LRNC may turn into fibrosis in months as a natural process. However, most previous investigations indicated that plaque seemed prone to progress rather than regress without lipid-lowering therapy [31,32], and LRNC was usually regard as one of the important factors leading to plaque increase. Secondly, as subjects were screened to have large plaques in this study, we are unable to discern whether or not the observed changes in LRNC volume and % LRNC occur in population with carotid wall thickness < 3 mm. Finally,due to the rigorous inclusion criteria and image quality requirement,a small number of subjects were enrolled and completed the trial. The degree to which lipid depletion documented by MRI will translate into an improvement in clinical outcomes remains unknown. Thus, larger randomized controlled trials are needed to confirm and further elucidate the effect of rosuvastatin on lipid-core reduction in patients with carotid atherosclerotic plaque in the future.

## Conclusions

In summary, the present study demonstrated rapid plaque lipid depletion with a moderate-dose rosuvastatin therapy among treatment naïve subjects with asymptomatic carotid lipid-rich atherosclerotic plaques. These findings may indicate an early on-set of plaque stabilization that could be achieved within the first three months of statin treatment.

## Abbreviations

MRI: Magnetic resonance imaging; LDL-C: Low-density lipoprotein cholesterol; HDL-C: High-density lipoprotein cholesterol; TOF: Time-of-flight; PDW: Proton-density-weighted; T2W: T2-weighted; T1W: T1-weighted; LA: Lumen area; WA: Wall area; PWV: Percent wall volume; LRNC: Lipid-rich necrotic core; CET1W: contrast enhanced T1-weighted.

## Competing interests

Authors received funding from AstraZeneca to carry out the MR imaging analysis. Other competing interests: none to disclose.

## Authors’ contributions

PY and LM designed the study and were involved in data analysis and interpretation. RD and JC contributed to data collection and interpretation. PY, XZ and RD wrote the manuscript. All authors were involved in the revision and approved the final manuscript.

## Pre-publication history

The pre-publication history for this paper can be accessed here:

http://www.biomedcentral.com/1471-2261/14/83/prepub
